# Solid Acid Resin Amberlyst 45 as a Catalyst for the Transesterification of Vegetable Oil

**DOI:** 10.3389/fchem.2020.00305

**Published:** 2020-04-29

**Authors:** Natalia Mariano Cabral, Juliana P. Lorenti, Winfried Plass, Jean Marcel R. Gallo

**Affiliations:** ^1^Group of Renewable Energy, Nanotechnology, and Catalysis (GreenCat), Department of Chemistry, Federal University of São Carlos, São Carlos, Brazil; ^2^Institute of Inorganic and Analytical Chemistry, Friedrich Schiller University Jena, Jena, Germany

**Keywords:** biodiesel, transesterification, ethanolysis, Amberlyst 45, acid catalysis

## Abstract

Commercial transesterification of vegetable oil to biodiesel using alkaline hydroxides requires expensive refined vegetable oil and anhydrous alcohols to avoid saponification. These issues are not present in the acid-catalyzed process; however, the challenge still lies in developing stable and active solid acid catalysts. Herein, Amberlyst 45, a resin for high-temperature application, was efficiently used for biodiesel production by the methanolysis or ethanolysis of vegetable oil. Yields of up to 80 and 84% were obtained for the fatty acid methyl ester and the fatty acid ethyl ester, respectively. Two processes are proposed and showed to be efficient: (i) incremental addition of alcohol along with the reaction for both methanolysis and ethanolysis; or (ii) one-pot reaction for ethanolysis using oil/ethanol molar ratio of 1/18. The catalytic system used also showed to be compatible with used oil (2.48 ± 0.03 mg_NaOH_ ${{\text{g}}_{\text{oil}}}^{-1}$) and to the presence of water (10–20 wt. % based on the alcohol), allowing the use of waste oil and hydrated alcohol.

## Introduction

The worldwide consumption of transportation fuels reached ca. 110 quadrillion BTU in 2015, and Diesel accounted for 21% (EIA, 2017). Compared to gasoline, Diesel exhibits higher efficiency and lower emission of greenhouses gases (GHG), outstandingly carbon dioxide (Heck and Farrauto, 2001). Due to the Paris Agreement and many other local actions for reducing CO_2_ emission (such as the European Climate action, EPA Regulations for Greenhouse Gas Emissions in the USA, and RenovaBio by the Brazilian CNPE), there is an increasing tendency of Diesel demand over other petroleum sources. For instance, the IMO's Marine Environment Protection Committee (MEPC) country members signed an agreement to reduce by 50% the CO_2_ emission by improving the use of marine Diesel (US EPA, OAR, OTAQ, 2018).

Several countries in the Americas, Europe, Asia; and also some states in the USA and Canada have mandates for biodiesel blends with Diesel (Lane, 2014; Scott and Sabine, 2018). In that matter, Brazil regulated the use of a 12% blend (B12), while Germany restrained GHG emission by blending biofuels, which led to a blend of 5–6% of biodiesel. Those actions promoted a systematic growth in biodiesel production, reaching 36 billion liters in 2017 (OECD and Food Organization of the United Nations, 2008-2017 (2009). A stable market for biodiesel is foreseen for the next decade, which motivates research for further development of the production process.

Commercially, biodiesel is synthesized by transesterification of vegetable oil with methanol, resulting in the desired fatty acid methyl ester (FAME) and glycerol. Sodium or potassium hydroxide are commonly used as catalysts, considering their low cost, high catalytic activity at low temperature (ca. 70°C), and high yield to FAME (Abdullah et al., 2017). However, there are major problems related to the homogeneous base-catalyzed biodiesel production. The most common is the saponification due to the production of the free fatty acid (FFA) salt, which not only reduces the catalyst concentration but also promotes the formation of an emulsion that hinders the glycerol separation. Soap is mainly formed by the hydrolysis of the esters to FFAs, followed by neutralization with the base catalysts (Pullen and Saeed, 2015). Since saponification is facilitated in the presence of water, anhydrous alcohol must be used (Sani et al., 2014; Su and Yihang, 2014; Abdullah et al., 2017). While this might not be an issue for methanolysis, it limits the use of bioethanol, due to the cost of its anhydrous form. Indeed, in the context of future established biorefinery, bioethanol would be the ideal alcohol for biodiesel synthesis, as it is largely produced from biorenewable sources (Gallo et al., 2014; De Oliveira and Coelho, 2017).

In the past decades, the development of efficient heterogeneous catalysts for vegetable oils transesterification has been one of the major challenges in biodiesel production (Kouzu et al., 2008; Su and Yihang, 2014). For that reason, several different heterogeneous basic catalysts have been tested, showing promising results (Taufiq-Yap et al., 2014; Hernández-Hipólito et al., 2015; Liu et al., 2015; Wong et al., 2015). For instance, CaO-based catalysts are widely applied in the transesterification reaction (Granados et al., 2007; Baskar and Aiswarya, 2016; Maneerung et al., 2016; Marinković et al., 2016; Roschat et al., 2016), because of their low cost and high effectiveness (Baskar and Aiswarya, 2016). However, basic catalysts suffer from strong deactivation by atmospheric water and CO_2_, and therefore, need to be activated at 700°C prior reaction (Granados et al., 2007). Furthermore, CaO was shown to deactivate due to the leaching of surface CaOH species (Granados et al., 2007). Besides, the optimal methanol to oil molar ratio for the CaO catalysts was higher than the practical ratio of 6:1 for homogeneously catalyzed transesterification (Roschat et al., 2016) and as a result of the solubility of glycerol in alcohol, in a batch reactor, a vast excess of methanol increases the glycerol concentration in the mixture shifting the equilibrium to the reactants (Maneerung et al., 2016). Basic polymeric resins, such as Amberlite IRA96 (Rosa et al., 2015) and Dowex monosphere 550 A (Marchetti et al., 2007), was shown to be active for transesterification, however, they also require high alcohol/oil ratio, making the process unrealistic for large-scale application.

As mentioned before, processes using a basic catalyst, homogeneous or heterogeneous, suffer from saponification, require refined vegetable oil, and have a low tolerance to water. Hence, intense research has been made in replacing the basic catalysts by heterogeneous acid ones (Mansir et al., 2017). In general, solid acids are less active for transesterification, requiring higher temperature compared to the basic catalysis, furthermore, side reactions such as alcohol and glycerol etherification can take place (Sani et al., 2014). Their advantage, however, is related to the non-formation of soap and the compatibility with the presence of water and free fatty acids, so those acids catalysts can catalyze simultaneously esterification and transesterification reactions (Abdullah et al., 2017).

Mixed metal oxides and sulfated metal oxides have dominated the literature for solid acid catalysts to biodiesel production due to their easy preparation and low cost (Sani et al., 2014; Vasić et al., 2020). For instance, ZrO_2_/SiO_2_ showed good activity for the esterification of stearic acid at 140°C, however, the alcohol/acid molar ratio was too high (120:1) (Ibrahim et al., 2019). Another example is the sulfonated magnetic solid acid catalyst (ZrFe-SA-SO_3_H) that obtained high yields of biodiesel (>92%) at 90°C for 4 h but deactivated after the first catalytic cycle (Wang et al., 2019). For TiO_2_/propylsulfonic acid nano-catalyst, the activity for transesterification reaction depends on the stability of the catalyst and at high FFA concentration in the oil, the FAME yield decreases (Gardy et al., 2017). Many other inorganic materials, such as silicates and heteropolyacids have been studied, the first suffers from the low activity and the second from low stability (Mansir et al., 2017).

Within the commercial solid acids, the cation exchange resins have an important industrial application, for instance, in esterification reactions. Resins, such as Amberlyst 15, Amberlyst 16, Amberlyst 35, and Dowex HCR-W2 have been successfully used for the esterification of FFA (obtained from waste oil) with methanol at 60°C (Özbay et al., 2008). Other resins and sulfonated solids were also active for the esterification reaction (Tejero et al., 2016; Zhang et al., 2020). The use of Amberlyst 15 for simultaneous esterification and transesterification would only be applicable if the water generated in the reaction was removed from the reaction mixture, thus avoiding the hydrolysis of triglycerides (Boz et al., 2015). However, most of the organic resins have low thermal stability (<130°C), limiting their application for transesterification (Sani et al., 2014).

To address the resin thermal stability, Dow Chemicals released the Amberlyst 45, a macroporous sulfonic acid polymer catalyst particularly well-suited for processes such as esterification, olefin hydration, and aromatic alkylation at temperatures of up to 170°C (AMBERLYST^TM^ 45 Resin High Temperature Strongly Acidic Catalyst n.d.^1^). Although not yet reported, this resin could present adequate properties for application in transesterification. From those points of view, this work aims to study the viability of Amberlyst 45 for the direct transesterification of vegetable oils to biodiesel through methanolysis and ethanolysis, producing, respectively, fatty acidy methyl ester (FAME) and fatty acid ethyl ester (FAEE). Under optimized conditions, the effect of using waste oil and the effect of water is also reported for the reaction with ethanol.

## Materials and Methods

### Materials

Amberlyst 45 (Surface area: 49 m^2^/g; Average pore size:19 nm; acid site density: 2.95 mmol g^−1^). (AMBERLYST^TM^ 45 Resin High Temperature Strongly Acidic Catalyst n.d.) was obtained from Dow Water & Process Solutions. Before use, the resin was thoroughly washed with distilled water, dried at 110°C for 24 h and ground to a fine powder.

Refined corn oil (Tegut) (acidity 0.38 ± 0.01 mg_NaOH_ ${\text{g}}_{\text{oil}}^{-1}$) was purchased in a supermarket in Jena, Germany. Used oil (acidity 2.48 ± 0.03 mg_NaOH_ ${\text{g}}_{\text{oil}}^{-1}$) was obtained in an Asian Restaurant in the same city and it was dried and filtered before use. Anhydrous methanol (<0.05% of water) and ethanol (0.04% of water) were used for the transesterification reactions.

### Transesterification of Vegetable Oil With Methanol or Ethanol in a Batch System

In a typical procedure, the reaction was performed under stirring (350 rpm) at 150 or 170°C in an Ace Pressure tube reactor loaded with vegetable oil, alcohol (oil/alcohol molar ratio between 1/3 and 1/18) and the catalyst (loading between 5 and 15 wt. % respect to the oil). The total amount of the liquid reactants were always 2.00 g, despite the oil/alcohol ratio.

At the desired time (between 15 and 360 min), the reaction was stopped by cooling down the reactor in an ice bath. The recovery of the reactional mixture for analyses was carried out as described elsewhere (Garcia et al., 2008). The mixture was centrifugated and the catalyst was decanted and separated. The unreacted alcohol was removed under vacuum, the mixture was centrifuged, and the glycerin separated by decantation.

### Etherification of Glycerol With Ethanol

The reaction was carried out similarly to the described for transesterification, but vegetable oil was replaced by glycerol, in the same to obtain a glycerol/ethanol molar ratio of 1/6.

### Etherification of Ethanol

The reaction was carried out similarly to the described for transesterification except that vegetable oil was not added to the reaction.

### Stability Studies for Ethanolysis of Vegetable Oil

Catalyst stability studies were carried out by reuse of the catalyst in an oil/ethanol molar ratio of 1/18, at 170°C for 120 min using 10 wt. % of catalyst. After each reaction, the catalysts were washed with water and ethanol and dried overnight at 100°C.

### Quantification of the Reaction Mixture

The yields of methyl or ethyl ether were determined by ^1^H NMR. Spectra were recorded using a Bruker Avance III 400MHz NMR spectrometer. Approximately 15 mg of each sample was dissolved in 0.5 mL deuterated using a 5 mm NMR tube. The quantification has been carried out as previously proposed by Schuchardt and co-workers (Gelbard et al., 1995; Garcia et al., 2008).

Quantification of the fatty acid methyl ester (FAME) obtained in the methanolysis of vegetable oil was performed using a method described previously (Gelbard et al., 1995).

$$\begin{array}{l} Yield_{FAME}=\frac{2}{3}\frac{A1}{A2}\times 100 \end{array}$$

Where,

A1 = area of the methoxy hydrogens

A2 = area of hydrogen in the α-carbonyl CH_2_

Quantification of the fatty acid ethyl ester (FAEE) obtained by ethanolysis of vegetable oil was performed as previously reported (da Silva et al., 2015).

$$\begin{array}{l} Yield_{FAEE}=\frac{A3-A4}{A2}\times 100 \end{array}$$

Where,

A2 = area of hydrogen in the α-carbonyl CH_2_

A3 = area of hydrogens of the CH_2_ of FAEE ethoxy group and also two hydrogens of acylglycerols

A4 = area of other two hydrogens of acylglycerols

Ethanolysis of glycerol was performed at conversions below 30% in order to calculate the initial rate. The product yield was obtained by ^1^H NMR following the equation:

$$\begin{array}{l} Yield\left(glycerol-ethanol\;ether\right)=\frac{2A1}{A3+A4}\times 100 \end{array}$$

Etherification of ethanol to diethyl ether was quantified by high-performance liquid chromatography (HPLC) using a Shimadzu LC-10/20 chromatograph coupled to a Bio-Rad Aminex HPX-87H (300 × 7.8 mm) column and a refraction index detector.

## Results and Discussion

Since the solid acid-catalyzed transesterification of vegetable oil usually requires high temperatures, sulfonated resins are commonly not suitable for this reaction due to low stability (Abdullah et al., 2017). Amberlyst 45 has been introduced in the market by Dow Chemicals for catalytic application up to 170°C and hence has potential application in the biodiesel synthesis by direct transesterification. Although most of the studies on transesterification focus on the methanolysis (Ma and Milford, 1999; Meher et al., 2006; Melero et al., 2009; Su and Yihang, 2014; Abdullah et al., 2017), in the context of a future established biorefinery, bioethanol would be preferred.

**Figure 1:**
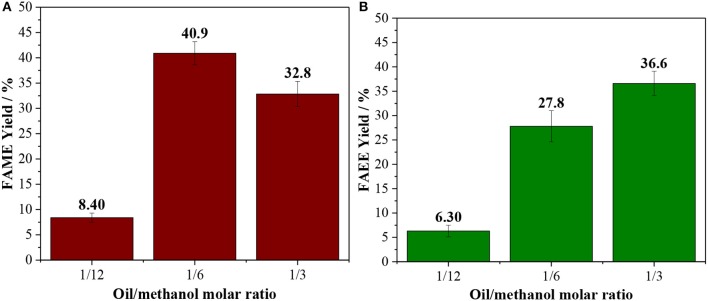
Effect of the oil/alcohol molar ratio [**(A)** methanol; **(B)** ethanol] in reaction performed at 150°C for 120 min using 10 wt. % of Amberlyst 45.

In order to establish the optimal operating conditions for reaction in batch systems, the oil/alcohol molar ratio and the catalyst loading were studied. The stoichiometric oil/ethanol molar ratio is 1/3, however, an excess of alcohol is commonly used to drive the reaction to high yields (Verma and Sharma, 2016; Andrade et al., 2020). Both methanolysis and ethanolysis were studied with an oil/alcohol ratio between 1/3 and 1/12. At low reaction times (60 min), the lower is the alcohol loading the higher is the yield for biodiesel (Figure S1), however, at longer reaction times (120 min), the experiment carried out with oil/alcohol molar ratio of 1/6 led to higher yields (ca. 41%) compared to 1/3 (Figure 1). These results endorse the importance of using an excess of alcohol. Interestingly, if the alcohol content is increased further to a 1/12 mixture, the yields drop to below 15%, due to a less effective dispersion of the catalyst, leading to mass transfer issues.

Previous studies using ion-exchange resin as the catalyst for the conversion of vegetable oil and other bulk substrates (Chakrabarti and Sharma, 1993; Tejero et al., 2016; Soto et al., 2018) show that external diffusion does not control the rate of reaction, unless the reaction system is very viscous or the agitation speed is insufficient, but poor dispersion of the catalyst can lead to a reduction in the activity (Chakrabarti and Sharma, 1993). Indeed, Amberlyst 45 is a macroreticular resin (Guilera et al., 2015) and, hence, it presents porosity independent on swelling (Ramírez et al., 2017). In swollen sate, macroreticular resins show three types of pores: non-swelling micropores, new mesoporous, and macroporous coming from permanent porosity. Therefore, the catalytic activity of macroreticular resins is effective in both swelling and non-swelling medium (Ramírez et al., 2017). For instance, dry Amberlyst 70 showed good swelling capacity when gamma-valetolactone is used as the solvent, leading to improvement in the reaction rate (Ramírez et al., 2017). As for the conversion of vegetable oil, methanol and ethanol were shown to have swelling rates similar to water in Amberlyst 15, and hence, these alcohols are expected to promote the swelling of Amberlyst 45 (Cabrera-Rodríguez et al., 2017; Soto et al., 2018).

**Figure 2:**
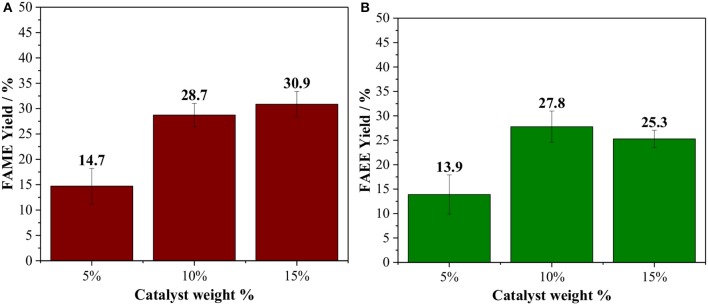
Effect of the Amberlyst 45 weight % based on the oil loading, reaction performed at 150°C for 60 min using oil/alcohol ratio of 1/6 [**(A)** methanol; **(B)** ethanol].

The effect of the catalyst loading was studied between 5 and 15 wt. % based on vegetable oil (Figure 2). By raising the catalyst loading from 5 to 10 wt %, the biodiesel yields, as expected, increase by two-fold for both methanolysis and ethanolysis. No significant increase in the product yields is observed by using 15 wt. % of Amberlyst 45, which could suggest that diffusion starts to limite the reaction rate under this condition, due to an inefficient dispersion of the catalyst caused by a large amount of the solid phase. Hence, catalyst loading of 10 wt. % and oil/alcohol molar ratio of 1/6 were chosen as the optimal parameters for Amberlyst 45.

**Figure 3:**
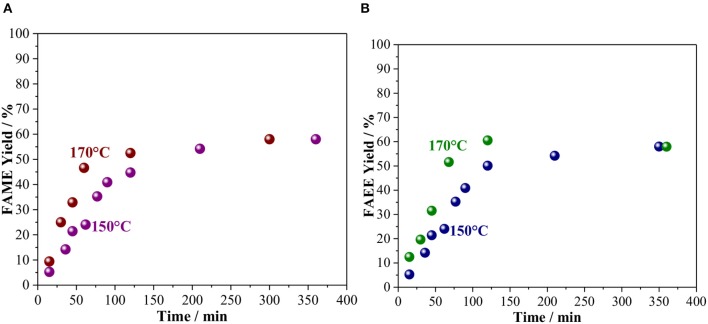
Kinetic curve of vegetable oil transesterification with **(A)** Methanol and **(B)** Ethanol catalyzed by Amberlyst 45 at 150 and 170°C. Reaction conditions: 10 wt. % of the catalyst based on oil and oil/alcohol ratio of 1/6.

Using the optimal conditions established above, alcoholysis of vegetable oil was studied as a function of time and the kinetic curves are shown in Figure 3. At 150°C, the product yield increases along with the reaction time and stabilizes at ca. 55%. Increasing the temperature to 170°C did not affect the final yield, but the time for reaching a maximum yield was two times lower than the reaction at 150°C, for both alcohols. It is indeed interesting that for both methanol and ethanol the initial rate of the reaction is very similar, as observed by the slope of the curve mol of the product as a function of reaction time (Figure S2). Previous work, indeed, shows that the reaction rate of ethanolysis and methanolysis of vegetable oils are comparable (Verma and Sharma, 2016). However, at 120 min, when the biodiesel yield stabilizes, FAEE is obtained with a yield slightly higher than FAME, 60.6 and 52.5%, respectively.

Two hypotheses were raised to explain the apparent interruption of the reaction observed for the reaction with both alcohols: (i) catalyst deactivation; or (ii) consumption of the alcohol due to side reactions. Indeed, ^1^H NMR and HPLC analyses revealed the presence of dimethyl ether or diethyl ether and ethers formed by the reaction between glycerol and the alcohol. Ethers are commonly pointed out as major co-products in acid-catalyzed transesterification of vegetable oils (Abdullah et al., 2017). The formation of these co-products can be taken in advantage, since they have been proposed to be used as additives to biodiesel (Bhide et al., 2003; Pinto et al., 2016; Sezer, 2019).

Related to the quantification of these co-products, the analytical methods used did not allow direct quantification with accuracy. Hence, ethanol etherification to diethyl ether and glycerol etherification with ethanol, were individually studied to determine their turnover frequency (TOF) and compare it to the TOF for FAAE formation. As shown in Figure S3 and Table S1, the TOF for FAEE formation is lower than those found for the formation of diethyl ether and the ethyl ether of glycerol. These results confirm that the transesterification of vegetable oil competes with etherification reactions, leading to the consumption of the alcohol, which justifies the stabilization of the FAME and FAEE yields observed in Figure 3.

**Figure 4:**
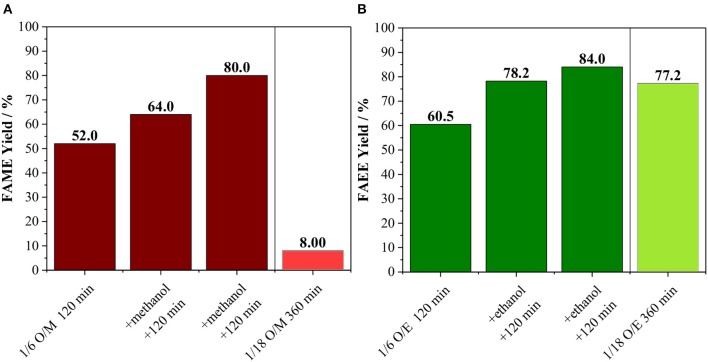
**(A)** Methanolysis and **(B)** Ethanolysis of vegetable oil using initial oil/methanol of 1/6 with the incremental addition of methanol every 120 min and comparison with the reaction performed with oil/methanol of 1/18 for 360 min. Both reactions carried out at 170°C with 10 wt. % of Amberlyst 45 based on oil loading.

Besides the conversion of alcohol into co-products, we also raised the possibility that the FAME and FAEE yield does not surpass ca. 60% due to catalyst deactivation. In order to confirm this hypothesis, an experiment with the incremental addition of alcohol was carried out (Figure 4). In this case, reactions were carried out for 120 min at 170°C using an oil/alcohol molar ratio of 1/6. After completion of the reaction, extra alcohol was added (the same amount used at the beginning of the reaction) and the reaction proceeded for another 120 min. This process was repeated twice. As shown in Figure 4, every addition of extra alcohol led to increasing biodiesel yield for both methanolysis and ethanolysis, suggesting that the catalyst was still active as also shown in Figure S4. Hence, the interruption in the biodiesel production observed in Figure 3 can be attributed mainly to the consumption of the alcohols due to the side reaction.

For sake of comparison, instead of three sequential additions of alcohol, an experiment was carried out using the total amount of the alcohol at the beginning of the reaction, which corresponding to an oil/alcohol molar ratio of 1/18 (Figure 4). For the methanolysis, the biodiesel yield was only 8.0 against 80.0% obtained for the experiment involving sequential additions of methanol. It is important to mention that the liquid phase mixture is initially biphasic, i.e., there is the oil phase and the alcohol phase. Amberlyst 45 disperses well at low methanol loadings, while it tends to agglomerate as the methanol loading increases.

For the ethanolysis, the reaction using oil/ethanol with a molar ratio of 1/18 led to a product yield of 77.2%, which is comparable to the 84.0% obtained by incremental addition of the alcohol (Figure 4). Indeed, it appears that Amberlyst 45 disperses better in the biphasic system oil-ethanol than in the oil-methanol, allowing the reaction to proceed even at high ethanol loadings, although higher reaction times are needed if compared to the 1/6 mixture. Hence, the low yields in the methanol system is due to a practical reason, i.e., in experiments using oil/methanol ratios up to 1/6, Amberlyst 45 dispersed well in the liquid, however, when the methanol loading is increased, the resin segregated from the liquid mixture and agglomerated on the walls of the reactor, leading to severe diffusion issues. Therefore, from an applied point of view, transesterification of vegetable oil with methanol using Amberlyst 45 in batch reactors requires a process with the incremental addition of the alcohol. Contrarily, for ethanolysis of vegetable oil, a one-pot process appears to be advantageous, which would be desirable for large scale applications.

Comparing the methanolysis and ethanolysis reactions, the product yields are very similar using an oil/alcohol ratio of 1/6 (Figure 3) or yet performing the experiments with incremental addition on alcohol (Figure 4). In these cases, Amberlyst 45 disperses well in the biphasic reaction mixture and mass transfer shall not limit the reaction, as previously observed for Amberlyst 15 (Boz et al., 2015). For acid-catalyzed transesterification, commonly, high alcohol loadings are needed (Boz et al., 2015; Alves et al., 2018; Cabrera-Munguia et al., 2018; Bernardes Costa et al., 2019; Kurhade et al., 2019; Silva et al., 2019; Syazwani et al., 2019; Wang et al., 2019; Andrade et al., 2020), and under this condition, Amberlyst 45 appears to be a more adequate catalyst for ethanolysis than methanolysis (Figure 4). This can represent great advantages in countries with a large production of bioethanol, such as the USA and Brazil, which happen to be also the largest producers of biodiesel (Biofuel.org.uk, 2010; Gallo et al., 2014).

The high ethanol loading necessary for the ethanolysis using Amberlyst 45 may imply in an increase in the process cost. However, since the excess of alcohol is converted to ether and ether of glycerol, the success of this catalyst would depend on finding a market for the co-products or in their use as an additive in the composition of biodiesel. Indeed, glycerol ethers (Melero et al., 2012) and diethyl ether (Miller Jothi et al., 2008; Ibrahim, 2018) have been proposed as fuel additives for biodiesel. Importantly, the boiling points of diethyl ether is close to room temperature, and it can be separated from biodiesel by distillation, if necessary.

**Figure 5:**
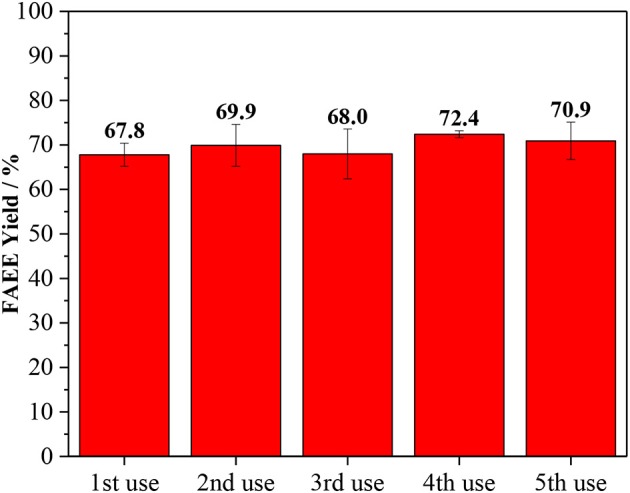
Recycling test for Amberlyst 45. Reaction conditions: oil/ethanol ratio of 1/18, the temperature of 170°C, 120 min and, 10 wt. % of catalyst loading based on the oil.

The stability of Amberlyst 45 was studied by multiple recycling experiments, as shown in Figure 5. The reusability of the catalyst is one important parameter for the feasible use in industrial scale. To examine that, subsequent transesterification reaction cycles were conducted at the optimum condition for ethanolysis (1/18 oil/ethanol, 10 wt. % of catalyst loading, 120 min and 170°C). After the reaction, the solid catalyst was recovered from the biodiesel product, washed with ethanol and dried before the reuse in the next cycle. Amberlyst 45 proved to be active after five cycles of use and the yield of biodiesel was not decreased.

For the acid-catalyzed ethanolysis of vegetable oil to make sense, the catalytic system must overcome the major problems associated with base catalysis: (i) the low tolerance to the presence of water, and (ii) the need of refined vegetable oil.

**Figure 6:**
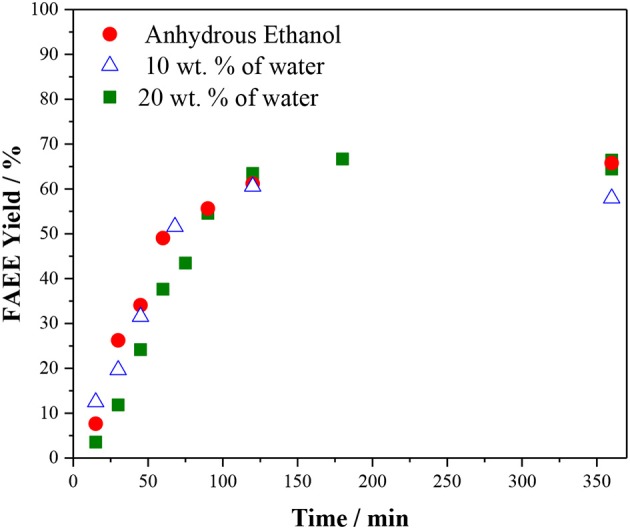
Effect of the water content in the vegetable oil ethanolysis. Reaction conditions: 10 wt % of Amberlyst 45, oil/ethanol ratio of 1/6 and temperature of 170°C.

It is important to remind that in the current commercial process the presence of water leads hydrolysis of triacylglycerol TAG, followed by the FFA neutralization with the base catalyst, producing soap. Therefore, for ethanolysis, anhydrous ethanol is needed implying in a prohibitive cost for biodiesel production. Hence, the effect of water was studied using ethanol containing 10 and 20 wt. % of water. As shown in Figure 6, there is no significant difference in the reaction performed using anhydrous ethanol and ethanol with 10 or 20 wt. % of water. This is indeed interesting since water is usually considered to deactivate acid resins (Buttersack et al., 1987) and even other types of acid catalysts (Xie and Hao, 2020). For the ethanolysis of vegetable oil catalyzed by Amberlyst 45, water affects very little the reaction rate and does not affect significantly the product yield. Therefore, hydrous ethanol (containing 8% of water, and ca. 30% cheaper than anhydrous ethanol) could be used in the reaction catalyzed by Amberlyst 45. Although Amberlyst 45 requires high temperature and ethanol loading to reach high biodiesel yields, it presents as an advantage the tolerance to the presence of water.

**Figure 7:**
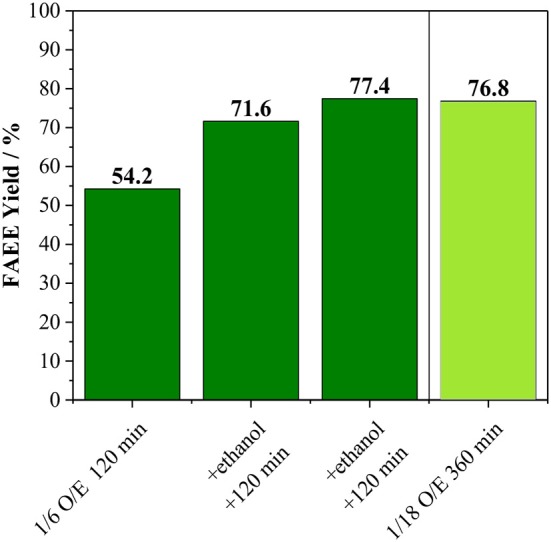
Ethanolysis of used vegetable oil using initial oil/methanol of 1/6 with the incremental addition of methanol every 120 min and comparison with the reaction performed with oil/methanol of 1/18 for 360 min. Both reactions carried out at 170°C with 10 wt. % of Amberlyst 45 based on oil loading.

The catalytic system was also tested in the transesterification of waste oil (used frying vegetable oil donated by an Asian restaurant). The acidity of this oil 2.48 ± 0.03 mg_NaOH_ ${\text{g}}_{\text{oil}}^{-1}$ (higher than the 0.38 ± 0.01 mg_NaOH_ ${\text{g}}_{\text{oil}}^{-1}$ found for the fresh oil, both calculated with the data on the Table S2), would cause problems of saponification and catalyst deactivation in the base-catalyzed commercial process. The acidity of waste cooking oil can vary depending on its nature, time and type of use (Cao et al., 2015; Park and Kim, 2016) and, in previous work, acidity was shown to vary between 2 and 6 mg_NaOH_ ${\text{g}}_{\text{oil}}^{-1}$(Cao et al., 2015; Park and Kim, 2016). Interestingly, using Amberlyst 45 as the catalyst, the ethanolysis of waste oil (Figure 7) led to FAEE yields similar to the ones obtained for commercial refined vegetable oil (Figure 4B) (for both oil/ethanol ratio = 1/6 with the incremental addition of ethanol and oil/ethanol ratio = 1/18).

Since Amberlyst 45 is compatible with used oil and hydrated ethanol, its use could contribute to reducing the cost of biodiesel production. Indeed, it has been shown that the prices of alcohol, catalyst, oil feedstock, and washing water are responsible for the largest percentage of the process operating cost (DiCosimo et al., 2013; Levett et al., 2016). Furthermore, recent studies show that for the FAME production at high pressure and temperature at supercritical (280°C and 28Mpa) and subcritical conditions (150°C and 20 Mpa), higher temperature and higher pressure do not increase significantly total energy consumption of the process (Glišić et al., 2009).

In comparison with other solid acid catalysts, Amberlyst 45 presents similar or superior performance. Furthermore, many studies on solid acids are carried out for the esterification of fatty acids instead of transesterification, and as it is shown herein, Amberlyst 45 is active in the direct transesterification. For instance, ZrO_2_ supported in Al_2_O_3_, Fe_2_O_3_, TiO_2_, or SiO_2_ is active in the esterification of stearic acid, but it requires (stearic acid)/alcohol ratio of 1/120 to reach 48.6% yield at 120°C (Ibrahim et al., 2019). Sulfonated magnetic solid acid catalysts reached 94% yield for biodiesel at 90°C, through the esterification of oleic acid, though, the catalyst partially loses activity after the first cycle (Wang et al., 2019). Sulfonated SBA-15 also led to over 90% yield for biodiesel from the esterification of oleic acid at 140°C and using (oleic acid)/methanol ratio of 1/15. The catalyst also displayed a decrease in the activity after the first use (Cabrera-Munguia et al., 2018). As for the direct transesterification of vegetable oils, sulfated zirconia reached over 90% yield for FAME at 200°C using an oil/methanol ratio of 1/6, however, it suffers from severe deactivation after the first use (Jitputti et al., 2006). Therefore, compared to other solid acid catalysts, Amberlyst 45 appears to be a promising catalyst since reaches ca. 80% yield for FAME or FAEE by direct transesterification of vegetable oil with an oil/alcohol ratio of up to 1/18.

## Conclusion

Amberlyst 45 was shown to be an efficient catalyst for transesterification of vegetable oil with methanol and ethanol at high temperatures (150 or 170°C) reaching FAME or FAEE yields of ca. 60% when using oil/alcohol ratio of 1/6. Higher yield of biodiesel (ca. 80%) could be obtained by incremental additions of the alcohol along the reaction and increasing the reaction time. Only for ethanolysis, the reaction using an oil/ethanol ratio of 1/18 in the one-pot regime also led to a high of FAEE (77.2% of FAEE after 360 min of reaction), which appears to be a great advantage over the process using incremental addition of alcohol.

The catalyst was also tested in conditions that are typically a problem in the actual commercial process: (i) use of frying vegetable oil feedstock with high acidity; and (ii) presence of high concentrations of water (10–20%). In both cases, FAEE yield was similar to the process using refined oil and anhydrous alcohol.

The advantages Amberlyst 45 compared to the commercial catalytic system using alkaline bases are: (i) absence of saponification; (ii) compatibility with low-refined vegetable oils and water; (iii) hydrated ethanol can be used; (iv) facile separation of the catalyst; (v) catalyst did not show deactivation after 5 cycles of utilization, confirming its stability under the reaction conditions; (vi) formation of valuable ether byproducts. The disadvantages of Amberlyst 45 are: (i) high reaction temperature; (ii) co-products, such as glycerol ether, need to be separated.

## Data Availability Statement

The datasets generated for this study are available on request to the corresponding author.

## Author Contributions

NC and JL carried out experiements. WP participated in the conception of the projects, co-advised the students, and wrote the manuscript. JG participated in the conception of the projects, ran experiments, advised the students, and wrote the manuscript.

## Conflict of Interest

The authors declare that the research was conducted in the absence of any commercial or financial relationships that could be construed as a potential conflict of interest.

## Footnotes

^1^*AMBERLYSTTM 45 Resin High Temperature Strongly Acidic Catalyst*. Available online at: http://www.dowwaterandprocess.com (November 4, 2018).
