# Supplementary material for: Solid Acid Resin Amberlyst 45 as a Catalyst for the Transesterification of Vegetable Oil
Source: Front Chem. 2020 Apr 29;8:305. doi: 10.3389/fchem.2020.00305 (PMC7201088; doi:10.3389/fchem.2020.00305)
Supplement: Supplementary file 1 [file Data_Sheet_1.PDF]

Supporting information for

Solid acid resin Amberlyst 45 as catalyst for the transesterification of vegetable oil

Natalia Mariano Cabral,[a,] Juliana P. Lorenti,[a,] Winfried Plass,[b] Jean Marcel R. Gallo[a,b,*]

[a]**G**roup of **Re**newable **E**nergy, **N**anotechnology, and **Cat**alysis (GreenCat), www.greencat.ufscar.br, Department of Chemistry, Federal University of São Carlos, Rod. Washington Luis, KM 235, CEP 13565-905, PO Box 676, São Carlos-SP, Brazil.

[b] Institute of Inorganic and Analytical Chemistry, Friedrich Schiller University Jena, Jena, Humboldtstr. 8, 07743 Jena, Germany.

*jean@ufscar.br

1. Effect of the oil/alcohol ratio at 60 min

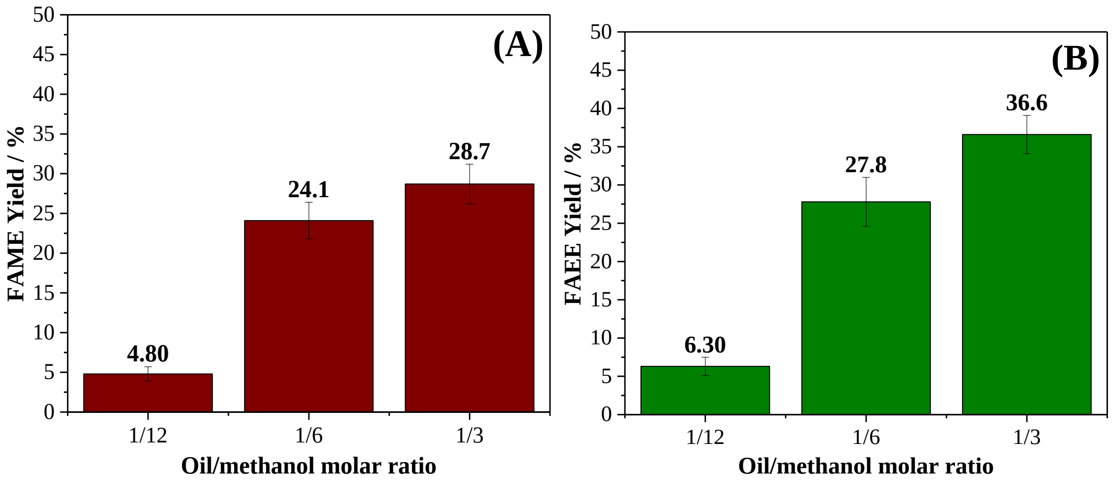

Figure S1. Effect of the oil/alcohol molar ratio [(A) methanol; (B) ethanol] in reaction performed at 150 °C for 60 min using 10 wt. % of Amberlyst 45.

2. Methanolysis vs. Ethanolysis

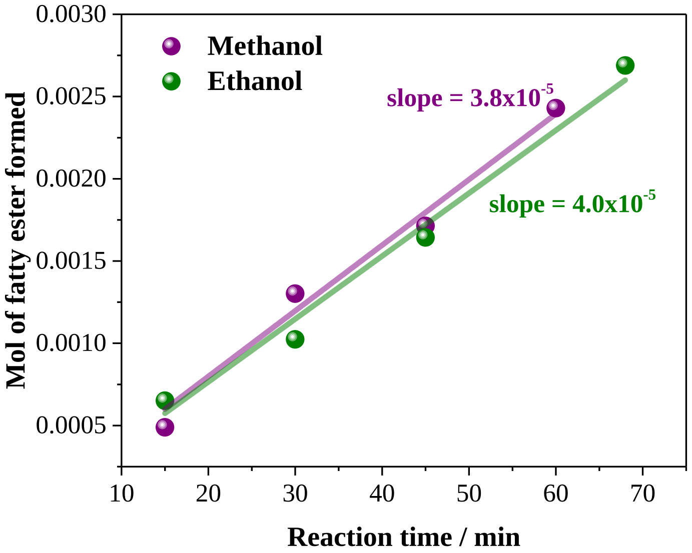

Figure S2. Fatty acid formation as a function of time for methanol and ethanol

3. Reaction of ethanol etherification

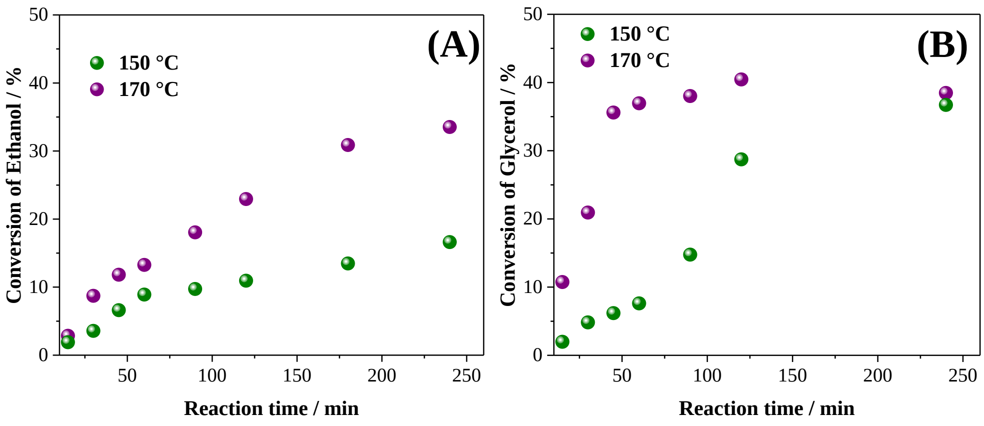

Figure S3. (A) Conversion of ethanol to diethyl ether and (B) Conversion of Glycerol by etherification with ethanol.

Table S1. Initial Turnover Frequency (TOF) for formation of FAEE, diethyl ether, and glycerol ethyl ethers.

Product	TOF / h^{-1}	
	**150 °C**	**170 °C**
FAEE[1]	1.1	4.1
Diethyl ether[2]	4.8	18.0
Glycerol ethyl ethers[1,2]	4.1	13.9

[1] oil/ethanol and glycerol/ethanol molar ratio of 1/6; [2] TOF for etherification reaction were determined individually;

Turnover Frequency (TOF) was given as:

$$\boldsymbol{TOF} = \frac{(\boldsymbol{mol\ ester\ formed})}{(\boldsymbol{catalyst\ weight})\boldsymbol{x}(\boldsymbol{acid\ sites\ by\ gram\ of\ catalyst})\boldsymbol{x}(\boldsymbol{reaction\ time})}$$

4. Stability test in fixed bed continuous flow reactor

4.1. Experimental setup for stability studies in a continuous flow reactor

Catalyst stability studies were carried out in a stainless-steel fixed-bed continuous flow reactor. Amberlyst 45 (3.00 g) was placed in the reactor between glass-wool plugs to avoid displacement of the catalyst bed. Two HPLC pump Jasco PU-2080Plus were used to feed the reactor with oil and alcohol with a total flow of 0.2 mL min^{-1} (oil/alcohol molar ratio of 1/6). The reaction was performed at 170 °C for 48 h.

4.2. Results

Although recycling the catalysts in batch reactors showed that Amberlyst 45 does not deactivate during the reaction, deactivation experiments in fixed bed flow reactor were carried out seeking a more quantitative result (**Figure S2**). A curve of ln(rate) as a function of time of stream was built in order to obtain the first order deactivation constant, which was 0.0256 h^{-1} for Amberlyst 45. For sake of comparison, Amberlyst 15, an acid resin with maximum operation temperature of 130 °C, was also tested and presented a deactivation constant 35 % lower. Indeed, Amberlyst 45 was not only more stable, but also more active, presenting an initial TOF of 0.2 h^{-1}, versus 0.12 h^{-1} for Amberlyst 15. Compared to the reaction in batch reactor, the use of Amberlyst 45 in the fixed bed continuous flow reactor led to a TOF 8.5 times lower (Erro! Fonte de referência não encontrada.). This suggests that problems of functional group leaching and/or diffusion could be present.

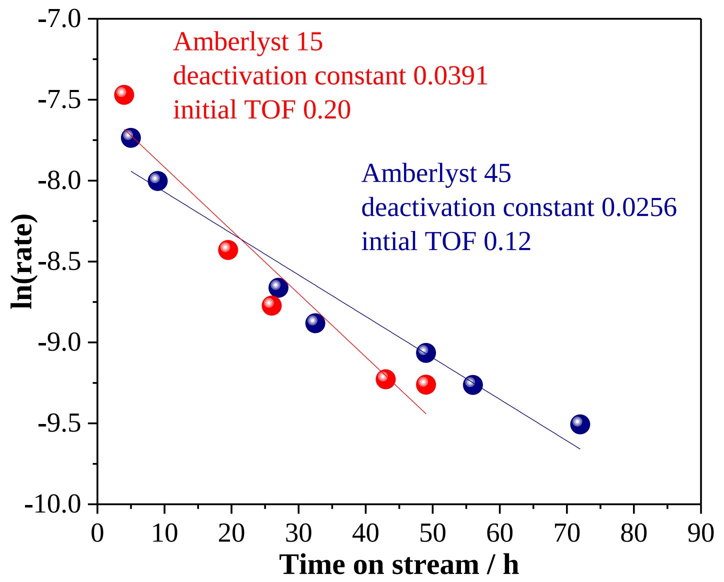

Figure S4. FAEE formation rate in logarithmic scale as a function of the time on stream. Reaction conditions: oil/ethanol ratio of 1/6 and temperature of 170 °C

5. Acidity of the fresh and used oils

The analyzes carried out following the ASTM D664 (2011).In a typical procedure, 2.0 g of oil were weighed in a 125 ml Erlenmeyer flask and 20 mL of neutral ether-alcohol solution (2:1 V:V) and two drops of the phenolphthalein indicator were added. The solution was titrated with a standard sodium hydroxide solution (0.0023 $molL^{-1}$ for fresh oil and 0.0102 $molL^{-1}$for used oil). The volume of base consumed in the titration is summarized in **Table S2**. The titrations were carried out in triplicate and the sodium hydroxide solutions were standardized.

Table S2. NaOH volume consumed and the mass of oil used in each titration

fresh oil			used oil		
Sample	Oil mass / g	V_{NaOH} / mL	Sample	Oil mass / g	V_{NaOH} / mL
1	2.04	8.10	1	2.01	12.15
2	2.07	8.75	2	2.07	12.80
3	2.04	8.30	3	2.01	12.10

The acidity was calculated as following the equation:

$$Acidity\ (mg_{NaOH}/\ g_{oil}) = \frac{\bar{v}_{NaOH}\ \ x\ C_{NaOH}\ x\ MM_{NaOH}}{m_{oil}}$$

Where:

v_{NaOH} = volume (L) of NaOH consumed in the titration;

C_{NaOH} = concentration (mol L^{-1}) of the standard NaOH solution;

MM_{NaOH} = molar weight of NaOH, 40.0 g mol^{-1};

m = weight of oil used for each titration.

Using the equation above, the acidity for the fresh and used oil was 0.38±0.01 and 2.48±0.03 $mg_{NaOH}\ g_{oil}^{-1}$, respectively.
